# Delayed Bilateral Vocal Cord Paralysis Following Cervical Spine Trauma

**DOI:** 10.7759/cureus.39891

**Published:** 2023-06-02

**Authors:** Jane Ehret, Andrew Thomas, David L Penn, Stanley Kaplan

**Affiliations:** 1 Internal Medicine, Vassar Brothers Medical Center, Poughkeepsie, USA; 2 Neurosurgery, Vassar Brothers Medical Center, Poughkeepsie, USA; 3 Pulmonary and Critical Care, Vassar Brothers Medical Center, Poughkeepsie, USA

**Keywords:** peripheral nerve disorders, surgical airway, neurology and critical care, critical care physician, otolaryngology-head & neck surgeons, blunt cervical trauma, acute care surgery and trauma, airway disorders, cervical spine fracture, bilateral vocal cord paralysis

## Abstract

Bilateral vocal cord paralysis is a potentially life-threatening condition, depending on the position in which the vocal cords are paralyzed. When the vocal cords are fixed in adduction, patients develop respiratory distress, inspiratory stridor, aspiration, and minimal phonation deficits. This condition can result from acute injuries to the right and left recurrent laryngeal nerves, or from chronic bilateral recurrent laryngeal nerve palsy. The clinical presentation is variable with such nerve injuries. Traumatic injuries to the cervical spine are an uncommon cause of this condition. In this report, we describe a patient who developed progressive respiratory distress, inspiratory stridor, and dysphagia to liquids several weeks after suffering major trauma to the head and neck. Laryngoscopy revealed immobile bilateral vocal cords fixed in the paramedian position, resulting in severe airway obstruction that warranted an emergency tracheostomy.

## Introduction

Vocal cord paralysis (VCP) is caused by the reduced or absent function of the vagus nerve or its branch, the recurrent laryngeal nerve (RLN). In contrast to unilateral VCP (UVCP) where the glottic aperture remains patent, it can become narrow when both sides are affected [1]. Depending on the position in which the vocal cords are paralyzed, patients with bilateral VCP (BLVCP) present with varying degrees of respiratory distress, inspiratory stridor, aphonia, dysphagia, and aspiration [1,2]. The incidence of BLVCP comprises about one-third of all VCP cases. Compared with UVCP, where normal ventilation can be relatively spared, the classical presentation of patients with BLVCP is a reduction of the glottal area resulting in various degrees of airway compromise. Patients will have noisy inspiratory breathing primarily with minimal voice change. BLVCP can be caused by iatrogenic injury with anterior neck surgeries, prolonged endotracheal intubation, tumors of the neck and thorax, brainstem strokes, neurodegenerative or demyelinating diseases, systemic inflammatory or infectious conditions, intrinsic vocal cord pathology, and traumatic injuries to the head and neck [3]. Non-surgical trauma is the least common with only a handful of cases reported in the literature [4-9].

Tracheostomy is one of the most common surgical interventions for BLVCP. Despite its effectiveness, tracheostomy is now becoming less favored by most patients because it presents an open wound that requires long-term care and creates psychosocial problems. Patients experience decreased quality of life and must engage in continual postoperative management of their tracheostomies. Recent evidence has shown tracheostomy is less cost-effective compared to endoscopic techniques (i.e., cordotomy and arytenoidectomy) in the management of BLVCP. Nevertheless, tracheostomy is still indicated as an effective, emergent, and initial method for the management of BLVCP over the short term.

We describe a patient who developed progressive respiratory distress, inspiratory stridor, and dysphagia to liquids several weeks after suffering major trauma to the head and neck.

## Case presentation

**Figure 1:**
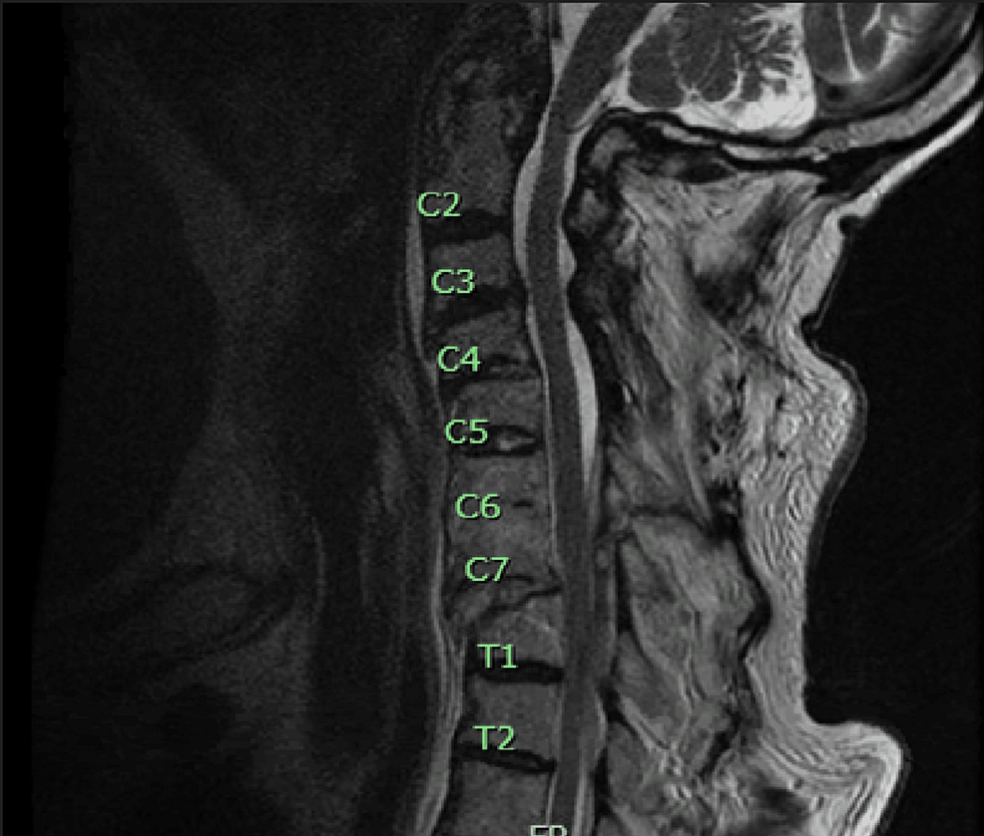
Sagittal T2-weighted MRI demonstrating an acute compression fracture of the superior endplate of T1 vertebra with surrounding edema, an unstable C7-T1 fracture with anterolisthesis, and a dorsal epidural hematoma from C5 to T2.

An 80-year-old male with a past medical history of atrial fibrillation on Coumadin, multilevel lumbar spinal canal, and neural foraminal stenosis presented to the hospital after falling down a flight of stairs. He was found to have a three-column fracture through multiple elements of C6, an unstable C7-T1 fracture of an anterior bridging osteophyte with resultant anterolisthesis, an acute T1 compression fracture with surrounding edema, and a large dorsal epidural hematoma extending from C5 to T2 (Figure 1).

Despite the extent of his trauma, he had no immediate neurological deficits. Posterior spinal fusion, laminectomy, and hematoma evacuation were performed without complication.

One month later, he returned with one week of worsening dyspnea, inspiratory stridor, and dysphagia to liquids. He was afebrile with a heart rate of 122 beats per minute, respiratory rate of 24 breaths per minute, blood pressure of 176/76 mmHg, and oxygen saturation of 100% on room air. His physical examination was significant for acute respiratory distress, audible inspiratory stridor, and no neurological deficits. Racemic epinephrine and IV dexamethasone were administered without clinical improvement.

Complete blood cell count and complete metabolic panel were unremarkable. Chest X-ray showed no evidence of acute cardiopulmonary disease. CT head showed no acute intracranial pathology. An urgent bedside flexible laryngoscopy revealed immobile bilateral vocal cords fixed in a paramedian position, causing severe glottic narrowing and salivary pooling in the hypopharynx. No edema, erythema, or masses were observed. On physical examination, the patient was able to talk in full sentences without any respiratory distress or pooling of saliva; however, his phonation was noted to be raspy in nature. Subsequently, he was taken to the operating room for an emergent tracheostomy.

Once his airway was secured, an extensive workup was performed to determine the etiology of his condition. No rheumatological, infectious, or central neurological causes were identified. There was no evidence of posterior glottic stenosis on CT imaging or with intra-operative palpation of the arytenoids. Anesthesia reports from his prior intubations showed no indication of traumatic intubation. It was concluded that his BLVCP was a delayed sequela of the extensive head and neck trauma he suffered one month earlier.

**Figure 2:**
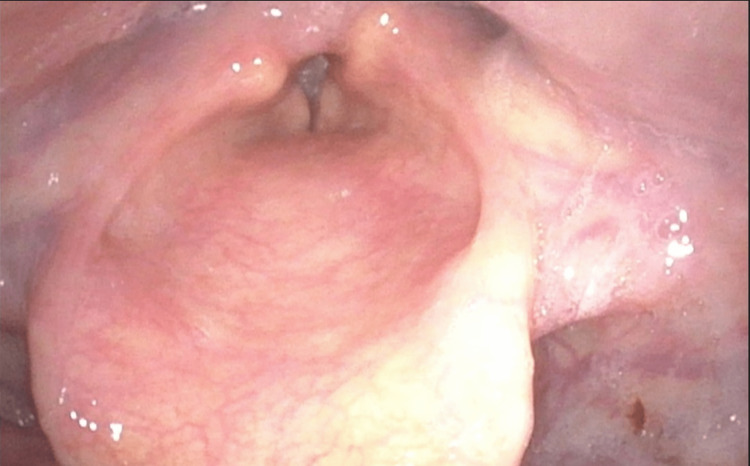
Laryngoscopy showing the vocal folds during phonation (speaking "eeee")

**Figure 3:**
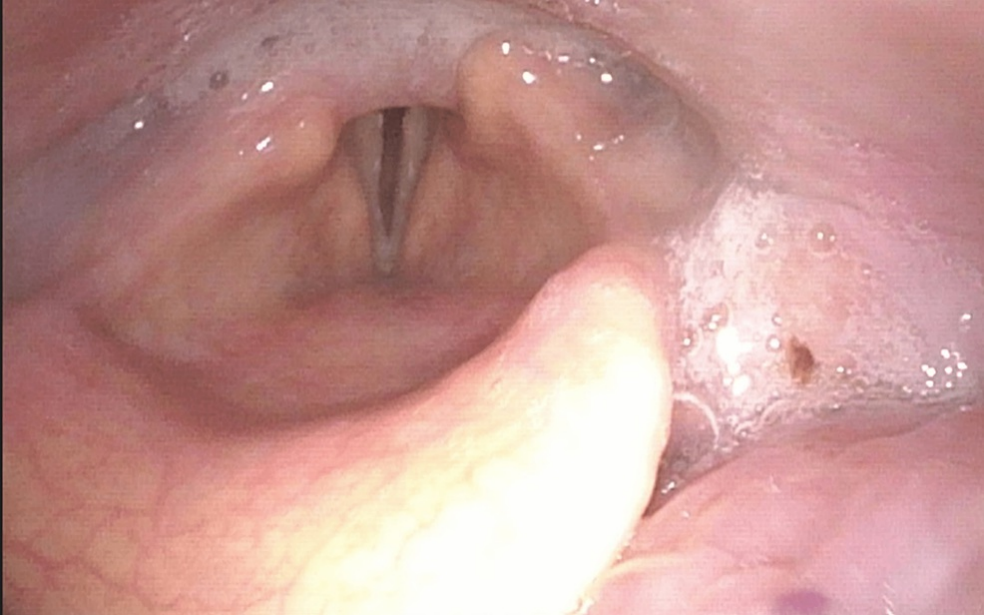
Laryngoscopy demonstrating the vocal folds at rest. The right arytenoid is in the medial position, while the left arytenoid is slightly more lateral, allowing for a small glottic gap. Salivary pooling is also seen.

The patient was transitioned to a tracheostomy collar and discharged to an acute rehabilitation center on hospital day 12. A repeat laryngoscopy was performed six weeks later, demonstrating persistent BLVCP (Figures 2, 3). 

His tracheostomy was ultimately removed nine weeks later. His respiratory status was stable after decannulation. After several months, he passed away at home from unknown causes. 

## Discussion

Respiratory failure in the setting of cervical spinal trauma typically results from diaphragmatic paralysis with C3-C5 injuries [10]. This case highlights the potential for respiratory failure with lower cervical spinal injuries that damage the RLN. Posterior glottic stenosis is another possible cause of BLVCP after trauma. The anatomy of the RLN as it relates to the cervical spine is key in this patient’s pathology, as well as identifying other trauma patients at risk for this complication.

Although the right and left RLN initially traverse separate courses after branching from the vagus nerves, they ultimately share an anatomical course as they ascend the tracheoesophageal groove, entering the larynx behind the cricothyroid joint at around the C6-C7 spinal levels [2,3,11]. Haller et al. found that the RLNs travel together above the C7-T1 spinal levels in most patients, making them equally vulnerable to injury above this level [11]. Our patient’s unstable C6-T1 injuries would have been high enough to damage both RLNs, but low enough as to not induce diaphragmatic paralysis.

The RLN innervates most of the intrinsic laryngeal muscles, making it responsible for both adduction and abduction motion. The posterior cricoarytenoid is the sole abductor, opening the glottis during inspiration. The thyroarytenoid, lateral cricoarytenoid, and inter-arytenoid muscles control adduction, closing the glottis during expiration and phonation. Recurrent laryngeal nerve axons can be variably expressed between abductors and adductors of vocal cords. Contrary to the belief that the action of the cricoarytenoid is overpowered by many adductor muscles, recurrent nerve axons may sparsely innervate vocal cord adductors in a few cases. Also, cricothyroid, spared tensor, equivocally abducts and adducts vocal cords [2,3]. As a group, the adductor muscles are approximately four times stronger than the single abductor muscle. The unequal distribution of muscle fibers becomes apparent when mild-to-moderate injury to the RLN impairs abduction more than adduction. Bilateral injury to the RLN paralyzes the vocal cords in a median or paramedian position. Because the glottis cannot open during inspiration, patients present with respiratory distress, inspiratory stridor, and minimal voice changes [12].

When the RLN injury is more severe, both abduction and adduction are impaired, paralyzing the vocal cords in a more lateral position. Respiratory symptoms are initially absent in these patients. Instead, these patients present with a “breathy” voice and aspiration events because the vocal cords are unable to close with phonation or swallowing [1,3,12]. As the RLN regenerates, airway symptoms can arise due to aberrant reinnervation of the laryngeal muscles, or synkinesis [12]. In such a synkinetic larynx, contraction of abductor and adductor antagonists produces ineffective, unsynchronized, or even opposite movement of the vocal fold [12]. The adductors are preferentially reinnervated due to the greater number of available motor units, causing the vocal cords to gradually migrate toward the midline [12]. This is what we believe happened in our patient, who developed airway symptoms several weeks after his injuries. Acknowledgment of this delayed clinical manifestation of cervical spinal trauma is critical to identifying those at risk for this complication. Patients presenting with signs and symptoms of VCP should be evaluated by an otolaryngologist with bedside laryngoscopy. 

## Conclusions

BLVCP is a potentially life-threatening condition with a heterogenous clinical presentation. The degree of injury to the recurrent laryngeal nerves determines the position in which the vocal cords become fixed. When the vocal cords are paralyzed in adduction, patients present with respiratory distress and inspiratory stridor. Symptoms may arise immediately or over several weeks to months due to aberrant reinnervation of the recurrent laryngeal nerves and laryngeal synkinesis. Traumatic injury to the head and neck is a rare cause of this condition. Patients who sustain traumatic injuries should be monitored for airway obstruction, even after their injuries have seemingly been repaired. Tracheostomy is the treatment of choice to ensure adequate ventilation. Other options for treatment include arytenoidectomy, cordotomy, reinnervation, laryngeal pacing, functional electrical stimulation, botulinum toxin, gene therapy, and stem cell therapy. 
